# Associating Air Pollution with Cytokinesis-Block Micronucleus Assay Parameters in Lymphocytes of the General Population in Zagreb (Croatia)

**DOI:** 10.3390/ijms231710083

**Published:** 2022-09-03

**Authors:** Goran Gajski, Marko Gerić, Gordana Pehnec, Katarina Matković, Jasmina Rinkovec, Ivana Jakovljević, Ranka Godec, Silva Žužul, Ivan Bešlić, Ante Cvitković, Pascal Wild, Irina Guseva Canu, Nancy B. Hopf

**Affiliations:** 1 Mutagenesis Unit, Institute for Medical Research and Occupational Health, 10000 Zagreb, Croatia; 2 Environmental Hygiene Unit, Institute for Medical Research and Occupational Health, 10000 Zagreb, Croatia; 3 Teaching Institute of Public Health Brod-Posavina County, 35000 Slavonski Brod, Croatia; 4 Faculty of Dental Medicine and Health, J. J. Strossmayer University of Osijek, 31000 Osijek, Croatia; 5 Faculty of Medicine, J. J. Strossmayer University of Osijek, 31000 Osijek, Croatia; 6 Center for Primary Care and Public Health (Unisanté), University of Lausanne, 1011 Lausanne, Switzerland; 7 PW Statistical Consulting, 54520 Laxou, France

**Keywords:** air quality, particulate matter, general population, peripheral blood lymphocytes, genome instability, public health

## Abstract

Air pollution is recognized as one of the most serious public health issues worldwide and was declared to be a leading environmental cause of cancer deaths. At the same time, the cytokinesis-block micronucleus (CBMN) assay serves as a cancer predictive method that is extensively used in human biomonitoring for populations exposed to environmental contamination. The objective of this cross-sectional study is two-fold: to evaluate genomic instability in a sample (N = 130) of healthy, general population residents from Zagreb (Croatia), chronically exposed to different levels of air pollution, and to relate them to air pollution levels in the period from 2011 to 2015. Measured frequencies of CBMN assay parameters were in agreement with the baseline data for the general population of Croatia. Air pollution exposure was based on four factors obtained from a factor analysis of all exposure data obtained for the examined period. Based on the statistical results, we did not observe a significant positive association between any of the CBMN assay parameters tested and measured air pollution parameters for designated time windows, except for benzo(a)pyrene (B[a]P) that showed significant negative association. Our results show that measured air pollution parameters are largely below the regulatory limits, except for B[a]P, and as such, they do not affect CBMN assay parameters’ frequency. Nevertheless, as air pollution is identified as a major health threat, it is necessary to conduct prospective studies investigating the effect of air pollution on genome integrity and human health.

## 1. Introduction

Rising industrial and energy production, the burning of fossil fuels and biomass, as well as the rise in road traffic, contribute to urban air pollution worldwide. Therefore, cities are generally hotspots for air pollution, which represents a global health problem, especially in urban centres resulting in almost seven million premature deaths annually [1,2,3,4,5] and affects the quality of life by causing the exacerbation of asthma, as well as other respiratory and cardiovascular problems. At present, more than 50% of the world’s population lives in urban areas, and thus the scope of health problems is paramount. To counter this, the EU has set a goal to achieve air quality levels that could reduce such a negative impact on, and risks to, human health and the environment [6,7,8]. Industrial and urban exhausts are two main sources of air pollution; however, environmental air pollution depends not only on the source and quantities of emission, but also on several other factors, such as weather conditions, seasonal variations, topography, location, etc. Because of this, each city presents a unique system.

Urban air is a complex and variable mixture of many different chemicals [9,10,11]. Particulate matter (PM) consists of inhalable particles that may include several harmful compounds, such as polycyclic aromatic hydrocarbons (PAHs) and heavy metals [10,12]. Both, particle size and content play an important role in affecting human health. Particle-size fractions PM_10_ (suspended PM with an aerodynamic diameter < 10 μm) and especially the smaller ones (PM_2.5_ and PM_1_, with aerodynamic diameters less than 2.5 and 1 μm, respectively) pose a significant health problem because they can penetrate the lungs and even the bloodstream [12,13,14,15,16,17,18,19,20,21,22,23,24,25,26]. For an estimation of population-weighted exposure to ambient particulate air pollution, the average levels of PM_2.5_ particle fraction concentrations are most commonly used, as this fraction is capable of penetrating deep into the lungs and causing severe, negative health effects [27,28,29]. However, the choice of fraction often depends on the availability of air pollution data. In particular, toxic airborne pollutants, such as PM, have been studied extensively concerning their effects on human health. The epidemiological studies conducted to date have observed a consistent association between exposure to airborne PM and incidence and mortality for lung cancer, respiratory, cardiovascular, and other disease [30,31,32,33,34,35,36,37,38,39,40]. The mechanisms of action for air pollution are not yet elicited, but oxidative stress and inflammation are suspected factors [40,41,42,43,44,45,46]. 

Cytogenetic methods play a crucial role in biomonitoring when evaluating the extent of chromosomal damage in human populations that have been exposed to different genotoxic agents. One of the best validated cytogenetic methods is the cytokinesis-block micronucleus (CBMN) assay [47,48,49,50,51,52,53,54,55] in peripheral blood lymphocytes (PBLs). It is frequently used in molecular epidemiology for the assessment of chromosomal damage resulting from exposure to environmental mutagens [51,56,57]. There are several biomarkers of genomic instability that can be measured using the CBMN assay. These are micronuclei (MNi), circular bodies made of chromosome fragments or whole chromosomes that indicate the downstream effect of DNA damage [58,59]. MNi, apart from representing the products of biological errors, such as mis-repair and/or replication errors, also indicates other biological effects. For instance, fragmented DNA in the form of MNi exposed to the cytoplasm may trigger the activation of immunity-system-related genes [60]. Furthermore, nucleoplasmic bridges (NPBs) originate from dicentric chromosomes caused by telomere end-fusions or the mis-repair of DNA breaks or failure of complete chromatid separation, while nuclear buds (NBUDs) are another indicator of chromosomal instability as they are a mechanism through which nuclei eliminate amplified genes and unresolved DNA repair complexes. Moreover, the assay can provide an index of cell proliferation and cytostasis that can be calculated from the nuclear division index (NDI) [47,58,59,61]. It has to be pointed out that elevated MNi frequencies in PBLs of healthy subjects have been shown to reflect an elevated risk of developing cancer later in life, suggesting a predictive role of this assay [62,63,64]. Lymphocytes are preferred for the measurement of genotoxic effects in biomonitoring studies since they present a measure of the total body burden of the genotoxicity and are considered early warning signs for adverse health effects [65,66,67]. 

In this cross-sectional study, we aim to associate historical air pollution measurements with the parameters of the CBMN assay in PBLs to evaluate possible effects of urban air pollution exposure on genome instability in Zagreb’s (Croatia’s) residents.

## 2. Results

### 2.1. Population Characteristics

This study included a group of 130 participants (89 females and 41 males) aged 19–80 years (average age, 38.08 ± 13.31 years; median age, 33 years) with a similar socio-economic status. Detailed population and lifestyle characteristics are presented in Table 1. All participants were selected from the general Croatian population and lived in the same region (Zagreb, the capital of Croatia), had similar patterns of physical activity, and similar levels of education (high school and university). None of the assessed subjects had been exposed to ionizing radiation, steroid therapy, or antibiotics for at least 3 months before blood sampling, as well as to occupational exposure that might have interfered with the results of the testing. Their mean body mass was 71.64 ± 14.29 kg, mean body height 1.72 ± 0.09 m, and mean body mass index (BMI) 23.99 ± 3.84 kg/m^2^. 

### 2.2. Baseline Frequency of the Cytokinesis-Block Micronucleus (CBMN) Assay Parameters

With the CBMN assay, the incidences of MNi, NPBs, and NBUDs were evaluated simultaneously with the cell-proliferation kinetics as measured by the NDI. The mean values and distributions of the CBMN assay parameters for the total population are summarized in Table 2. The mean MNi frequency for all of the subjects was 5.12 ± 2.82 per 1000 BNCs (median was 5), ranging from 1 to 13. The mean frequency of NPBs for all of the subjects was 1.22 ± 1.51 per 1000 BNCs (the median was 1), ranging from 0 to 7. The mean frequency of NBUDs for all of the subjects was 3.45 ± 2.10 per 1000 BNCs (the median was 3), ranging from 0 to 10. As for cell proliferation, the mean NDI was 2.00 ± 0.12, ranging from 1.60 to 2.38. 

### 2.3. Air Pollution Exposure

The mean exposure ± standard deviation and range for each measured pollutant are presented in Table 3. Among the PM_10_ constituents, the highest mean concentrations had organic carbon (OC) (8.16, 8.33, and 8.74 µg/m^3^ for 3, 7, and 30 days before blood sampling, respectively), followed by sulphate and nitrate, while the lowest mean concentrations were obtained for Cd (0.217, 0.222, and 0.229 ng/m^3^) and As (0.554, 0.569, and 0.562 ng/m^3^). For none of the pollutants did the mean log-transformed values differ significantly for different time windows. 

### 2.4. Influence of Air Pollution on the Cytokinesis-Block Micronucleus (CBMN) Assay Parameters

Based on the statistical analysis, we did not observe significant association between any of the CBMN assay parameters’ (total number of MNi, NPBs, NBUDs, and NDI) tested and measured air pollution parameters for the designated time windows of 3, 7, and 30 days before blood sampling. The results are presented in the Supplementary Materials (Supplementary Tables S1 and S2).

## 3. Discussion

During their daily activities, people are exposed to various environmental pollutants that may have cyto/genotoxic properties. This particularly concerns urban air, which is a complex mixture of different physical and chemical agents that possess mutagenic and carcinogenic properties, such as heavy metals, diesel soot, volatile organic compounds (VOCs), and PAHs [68,69,70,71,72,73,74,75]). In line with that, human biomonitoring is a critical instrument that allows us to evaluate to what extent different environmental substances affect the human population, providing valuable information on environmental exposure and helping us identify potential health risks. One of the strengths of human biomonitoring is that it can produce precise information for the overall exposure of an individual at a particular time point, as it adds together exposure from various sources and routes. Nonetheless, the risks these exposures may pose to human health, in which combination, and at what levels is quite challenging to estimate [67,76,77,78,79,80,81,82,83]. Therefore, we wanted to retrospectively associate air pollution with CBMN assay parameters in PBLs of the general population in the capital of Zagreb, which is the largest and the most populated city in Croatia. 

Previous studies of air quality in continental Croatia have shown that ambient air is polluted with PM and PAHs, especially during the colder part of the year (heating season) [84,85,86,87,88]. Levels of ambient air pollutants measured in Zagreb during this study did not differ significantly from values obtained in earlier investigations [85,87,89]. Similar or slightly higher benzo(a)pyrene (B[a]P) concentrations were observed in some other European urban areas, such as Zaragoza and Monagrega in Spain [90] and Naples, Italy [91], while higher concentrations were measured in Sarajevo, Bosnia and Herzegovina [92] and some rural residential areas in Germany during the winter season [93]. In Zagreb, levels of PM_10_ and PM_10_-bound B[a]P exceeded the limit and target values set by Croatian and EU legislation [7,8,94]. On the other hand, levels of toxic metals (Pb, As, Cd, Ni) were much lower than the regulatory limits [95,96], and were also comparable with previously reported ones. Zink levels reported in this study follow up on the decreasing trend of annual values for zinc in Zagreb air [97]. A similar decreasing trend was also pronounced for lead, manganese, cadmium, and nickel in PM_10_ from 2006 to 2011, while concentrations of arsenic were at similar levels as those reported here [95]. The ambient air levels of metals observed in this study were lower than the ones obtained from urban and urban background sites in some other studies [98,99,100,101,102,103] and similar to the concentrations of metals obtained from an urban background site in Italy [104].

Earlier Croatian legislation [105] set a limit value for sulphate in PM_10_ (30 µg/m^3^ for 24 average, 20 µg/m^3^ for annual average); however, as Croatia joined the European Union in 2013, the new regulation was adjusted with EU directives and limit values for acidic ions in the PM being omitted. In the new regulation, the measurements of water-soluble anions and cations, as well as organic and elemental carbon in PM_2.5_ particle fractions, are still required, but only to ensure adequate information for the comparison of background and more polluted areas, assess the possible contribution of the long-range transport of air pollutants, and support source-apportionment analysis and modelling. At present, for OC and elemental carbon (EC) there are no regulatory limit values, but these data may produce valuable information on possible pollution sources, such as traffic, biomass burning, or secondary (aged) aerosols [106,107,108]. Previous measurements of acidic anions in Zagreb have shown that the annual average mass concentrations followed the order of chloride < nitrate < sulphate. Over 7 years (1999–2005) the mean annual mass concentrations varied from 0.28 to 0.95 μg/m^3^ for chlorides, from 3.21 to 7.87 μg/m^3^ for nitrates, and from 3.98 to 9.71 μg/m^3^ for sulphates. The annual average mass ratio of NO_3_^−^/SO_4_^2−^ suggested that mobile source emission was an important contributor to particle mass [109,110]. Previous measurements of OC and EC in Zagreb have shown that EC ranged between 0.5 µg/m^3^ and 2.2 µg/m^3^ depending on particle size, location, and period of sampling, while OC ranged between 5 µg/m^3^ and 33 µg/m^3^ [85,108,111,112].

The factor analysis conducted in this study (described in Section 4.5) extracted 4 factors: a PM factor, 2 metal factors (F2–Mn, Cu, Fe, Zn, and F4–Pb, Cd, As, Zn), and the last F3 factor correlated to NO_3_^−^, Cl^−^, OC, EC, and B[a]P—negatively with SO_4_^2−^. As the PM_1_ particle fraction is part of PM_2.5_, and PM_1_ and PM_2.5_ are both part of PM_10_, they usually correlate well with each other, which can explain F1. Metal factors containing Zn, Cu, Fe, and Mn can be attributed to non-exhaust traffic emissions, which included additives to oil, tyre, and brakes wear [113,114]. Since Zn is often suggested as a marker for different sources, it is not uncommon to be found in the second factor with Pb, Cd, and As, which could be a mixed anthropogenic source, both from industrial and traffic emissions. Levels of NO_3_^−^, Cl^−^, OC, EC, and B[a]P in Zagreb are usually higher during the colder part of the year [85,86,87,109,110,115], while SO_4_^2−^ did not show a clear seasonal trend [110]. OC, EC, and B[a]P from F4 could originate from different combustion processes; during the cold part of the year, they could indicate biomass burning, probably wood-burning households, while during the warm period of the year, this factor could also represent traffic [108]. Elevated NO_3_^−^ levels could be due to the combined action of various factors, such as photochemical and heterogeneous reactions and emissions of NO_x_. The studies conducted by Jiang et al. [116] and Tao et al. [117] showed that low temperatures favour the conversion of ammonium nitrate and inhibit the secondary transformation of SO_4_^2−^. Cl^−^ could result from winter road salting or a tracer of coal combustion [118,119]. 

Based on the statistical results, we did not observe a significant association between any of the CBMN assay parameters tested and the measured air pollution parameters for the designated time windows in the period between 2011 and 2015, except for B[a]P that showed a significant negative association. This may be due to the fact that, in winter (when B[a]P levels are higher), people are mostly inside and are not exposed to measured B[a]P levels, and in summer (when B[a]P levels in the air are very low) we have some other factors (such as intense sunlight, solar radiation, high temperature, and high UV index) that can cause an increase in the measured parameters. Furthermore, in the summer months, PAHs (including B[a]P) are subjected to the reactions of photooxidation and oxidation with the ozone [120,121]. The degradation products of PAHs are complicated and depend on the propagation of the free-radical reaction in the atmosphere, potentially enhancing the ozone and UV-induced carcinogenesis by PAH compounds. Previous studies showed that PAHs can adsorb sunlight (visible and UV regions of the solar spectrum) causing structural modification and photoproducts, such as quinine, which were confirmed as toxic [122,123]. It was also shown that UV significantly impairs B[a]P metabolism, and decreases rather than increases immediate toxicity. However, it cannot be excluded that decreased metabolism leads to the accumulation of B[a]P and delayed genotoxic effects [124,125,126]. Nevertheless, air pollution’s effect on CBMN assay parameters in PBLs was noticed in several European cities and regions [56,127,128,129,130,131,132] as well as on the world scale [133,134,135,136,137,138,139] with outdoor city workers, such as traffic controllers, taxi drivers, and policemen, being at a greater risk. Children and newborns are also at a higher risk since they are far more sensitive to potentially mutagenic and carcinogenic effects of air pollution compared to adults. This risk is caused by children’s anatomical features and their longer life expectancy in which to express that risk. Nonetheless, there are studies indicating that exposure to urban air pollutants does not result in increased levels of MNi in peripheral blood cells or that the results are inconclusive [57,138,140,141,142]. Additionally, the above-mentioned studies provide evidence that the CBMN assay could be used as a sensitive indicator to air pollution-induced genotoxic effects in humans and favours it application in the studies of this type. Since MNi are biomarkers of early genetic effects that have been used to investigate the association between environmental exposures and cancer [62,63,64], these findings suggest that long-term exposure to air pollution could induce cumulative DNA damage, supporting the hypothesis that prolonged exposure to air pollution can contribute to cancer development posing a serious burden to the health system [143]. 

In addition to PM that was measured in this study, nitrogen dioxide and ground-level ozone are generally recognized as the pollutants that most significantly affect human health [9] and should also be included in the future studies when evaluating the impact of air pollution on genomic instability and human health. According to the EEA, 18 of the 28 EU countries in 2015 registered ozone concentrations greater than the human health protection target value (120 μg/m^3^), while the guideline value set by the World Health Organization (WHO) (100 μg/m^3^) was exceeded in 96% of all the reporting stations. The annual limit value for nitrogen dioxide was exceeded across the whole of Europe, with the highest values usually observed at traffic stations [6,11]. Their interaction with organic compounds, such as PAHs bonded to PM, may lead to the formation of some more dangerous compounds [120,121,144].

In summary, this study evaluated the possible associations between the CBMN assay parameters and historical air pollution data. One hundred and thirty subjects from the general population living in the city of Zagreb (Croatia) in the period from 2011 to 2015 participated in the study. Atmospheric levels of B[a]P and PM_10_ slightly exceeded regulatory limits during the observed period. On the contrary, concentrations of toxic metals and other PM constituents were relatively low and comparable with other urban locations in the region. The measured frequencies of CBMN assay parameters (micronuclei, nucleoplasmic bridges, nuclear buds, and nuclear division index) were in agreement with the baseline data for the general population in Croatia. We did not observe statistically significant associations between any of the CBMN assay parameters and measured air pollution data. Our results show that the measured air pollution parameters are considerably below the regulatory limits and, as such, they do not affect the CBMN assay parameter frequencies. Nevertheless, the global trend of rising air pollution, especially in urban city areas, indicates the necessity for conducting prospective studies on the effect of air pollution on genome integrity and human health. However, our results should be interpreted considering the limits of the study, such as the potentially non-representative sample, selection bias, uncontrolled confounding or effect modifiers, as well as missing biomarkers of exposure. In future studies, more attention should be paid to include outdoor as well as indoor air-exposure assessments; the use of biomarkers of exposure, including more complementary biomarkers of effect; optimising the width of the selected time windows; air pollutants’ measurements in relation to the type of each biomarker; as well as the inclusion of more air-monitoring stations in larger cities. These retrospective data will serve as a historical record that may contribute to a better understanding of how air pollution may influence the frequencies of CBMN assay parameters and can later serve as a comparison for future prospective studies.

## 4. Materials and Methods

### 4.1. Study Sample and Participant Selection

This cross-sectional study was conducted on a group of 130 participants extracted from our database for the period between the years of 2011 and 2015. The participants were collected either during various ecogenetic studies aimed at assessing the values of biomarkers of exposure, effect and genome sensitivity in the general population in the Republic of Croatia, or during the continuous biomonitoring of populations of workers, in line with Croatian regulations, conducted in that period. All participants were selected from the general Croatian population (both sexes) living in Zagreb (capital of Croatia). The selection criteria were that they were healthy at the moment of blood sampling and interviews, occupationally unexposed, and had not been exposed to ionizing radiation, steroid therapy, or antibiotics for at least 3 months before blood sampling. All participants provided their written informed consent and completed a questionnaire intended to obtain demographic data, smoking habits, alcohol consumption, health status, family history of cancer, and prior or current exposure to diagnostic radiation, and medication that could interfere with the obtained results. Privacy of the data is guaranteed. The study was part of the projects approved by the Ethics Committee of the Institute for Medical Research and Occupational Health, Zagreb, Croatia.

### 4.2. Blood Sampling

Venous blood was collected at the Institute for Medical Research and Occupational Health (Croatia) into sterile heparinised tubes (Becton Dickinson, Franklin Lakes, NJ, USA) in the morning hours (between 8 and 10 a.m.) by highly trained medical professional. Subsequently, blood samples were handled in the same manner, being randomly coded, stored at 4 °C, protected from light, and processed as quickly as possible (not more than 4 h after the sampling).

### 4.3. Cytokinesis-Block Micronucleus (CBMN) Assay

The CBMN assay followed the protocol by Fenech [47] and previously described in detail [48]. For the culture setup, 500 μL of whole blood was added to RPMI 1640 medium (Gibco, Carlsbad, CA, USA) supplemented with foetal bovine serum (FBS; Gibco), phytohemagglutinin (Remel, Lenexa, KS, USA), and antibiotics (penicillin and streptomycin; Sigma, St. Louis, MO, USA), and incubated at 37 °C and 5% CO_2_ for 72 h. Cytochalasin-B (Sigma) was added to each sample at a final concentration of 6 μg/mL after 44 h of incubation in order to prevent cytokinesis, and the cells were harvested at 72 h. The lymphocytes were fixed in a methanol–acetic acid solution (Kemika, Zagreb, Croatia), air-dried, and subsequently stained with 5% Giemsa (Merck, Darmstadt, Germany). An optical microscope with a final magnification of 400× (Olympus CX41, Tokyo, Japan) was used for slide analysis. Every subject was analysed for a total number of MNi, NPBs, and NBUDs per 1000 binucleated cells, while the NDI was calculated on the same slides by counting 1000 cells. 

### 4.4. Variables Tested

In the present study, age, sex, BMI, and lifestyle factors recorded in the questionnaire, including smoking habit, alcohol consumption, physical activity, and medical status that could affect the number of tested parameters, were considered as potential confounding variables. Moreover, air pollution measurements according to the sampling period were also considered.

#### 4.4.1. Age, Sex, and Lifestyle Factors

Factors, such as age, sex, and BMI, of the selected population; several lifestyle factors, such as smoking habit, alcohol consumption, regular exercise (at least two times per week for at least half an hour); and medical status (family history of cancer) that could affect the number of tested parameters were considered. Participants were categorised as non-smokers or smokers. Individuals who had been smoking for at least one year prior to blood sampling were considered to be smokers. Participants were also categorised as non-alcohol consumers and alcohol consumers. Individuals who said that they consumed alcoholic beverages (beer, wine, or spirits) once a week were considered to be alcohol consumers. Participants were also categorised for the occurrence of cancer in their family (family members who had or have different types of cancer). 

#### 4.4.2. Air Pollution Measurements

In this paper, the air pollution data for the period from January 2011 to January 2015 were used. The 24 h samples of three different PM fractions (PM_10_, PM_2.5_, and PM_1_) were collected. In the PM_10_ particle fraction, the following constituents were determined: metals (Pb, Ni, Cd, As, Fe, Cu, Zn, Mn), B[a]P, water-soluble anions (chlorides, nitrates, sulphates), OC, and EC. 

The measuring site for air-quality monitoring was located at the Institute for Medical Research and Occupational Health (45°50′6.8″ N, 15°58′42.12″ E, 168 m a.s.l.) in the northern, residential part of Zagreb (Croatia). The measuring station is classified as an urban background station and it is part of the local air-quality monitoring network funded by the City of Zagreb. Over the years, in addition to standard, routine pollutants required by the air-quality legislation (PM_10_, PM_2.5_, B[a]P, Pb, Ni, Cd, As in PM_10_), different particulate matter fractions and their constituents have been determined. 

In this study, air pollution data were obtained from the oldest Zagreb air-quality-monitoring station, which is also the only one with a complete five-year dataset on PM_10_ composition (metals, OC, EC, PAHs, major anions), as well as on PM_2.5_ and PM_1_. Preliminary data analysis (data available at http://iszz.azo.hr/iskzl, (accessed on 1 July 2022)) showed that levels of PM_10_ did not significantly differ between most of the stations. Higher values were observed only in the southern part of the town, but correlated well with other locations. Therefore, in the absence of other air pollution data, the existing data can be considered sufficiently representative of the majority of the area.

##### Gravimetric Analysis

The 24 h samples of three different PM fractions (PM_10_, PM_2.5_, and PM_1_) were collected each day from January 2011 to January 2015 on quartz filters with low-volume gravimetric samplers (LVS 3 single-filter sampler, Sven Leckel Ingenieurbüro GmbH, Berlin, Germany) from about 55 m^3^ of air. The samples were collected on several parallel samplers because each type of pollutant required a separate filter for chemical analyses.

After sampling, mass concentrations of the PM fractions were gravimetrically determined on a microbalance (Mettler Toledo MX-5, Mettler-Toledo GmbH, Greifensee, Switzerland) according to the EN 12341:2006 and EN 14907:2005 standards. Before and after sampling, the filters were conditioned at a constant temperature (20 ± 1 °C) and relative air humidity (50 ± 5% RH) for 48 h prior to the first weighing and reweighing after the subsequent 24 h.

##### Metal Analysis

For the analysis of metals, particulate matter filter samples were digested with nitric acid (25% HNO_3_ *v*/*v*) in a high-pressure microwave digestion system (Ultraclave IV, Milestone, Sorisole, Italy) and diluted with deionised water. The samples were analysed using inductively coupled plasma mass spectrometry, ICP-MS (7500cx, Agilent Technologies, Santa Clara, CA, USA). Isotopes ^55^Mn, ^56^Fe, ^60^Ni, ^65^Cu, ^66^Zn, ^75^As, ^111^Cd, and ^206^Pb were selected and the integration time per point was 0.5 s for As and Cd, and 0.1 s for other analysed metals with three acquisition points per peak. Scandium, germanium, rhodium, and bismuth were added as internal standards. The ICP-MS spectrometer was tuned to obtain an oxide ratio and doubly charged ratio <1.5%. The analysis was made in helium (He) mode. The instrument operating parameters were optimized to minimize the interferences and maximize the sensitivity. Working standards (5% HNO_3_ *v*/*v*) were prepared from single-element stock solutions (1000 μg/mL, SCP SCIENCE) at eight level concentrations, and the calibration was performed every time before the sample analysis. The accuracy of the method was determined by preparing and analysing PM_10_-like reference materials NIST 1648a and ERM CZ120 in the same way as the collected samples. The recoveries for the analysed metals ranged from 87% to 108% for both certified reference materials [95,96].

##### Benzo(a)pyrene (B[a]P) Analysis

For the B[a]P analysis, filters were extracted with a solvent mixture of toluene (Merck) and cyclohexane (Merck) (7:3) in an ultrasonic bath for 1 h, separated from undissolved parts by centrifugation (10 min, 3000 rpm), evaporated to dryness in a mild stream of nitrogen at 30 °C, and then re-dissolved in acetonitrile (Merck) [87]. The analysis was performed using Varian Pro Star high-performance liquid chromatography (HPLC, Varian, Victoria, Australia) with a fluorescence detector, which was programmed at eight different steps of excitation (λ = 234) and emission (λ = 500) to provide optimal detection. The mobile phase was a mixture of acetonitrile and water, and the flow rate was 0.55 mL/min. For separation, a Varian stainless-steel Pursuit 3 PAH column (3 µm, 4.6 × 100 mm) was used. Laboratory and field blanks were processed in the same way as real samples. For quality control and assurance, the analytical standard (Supelco EPA 610 PAH mix) and certified reference material (CRM NIST 1649b, Urban dust) were used [87,88,145].

##### Organic (OC) and Elemental (EC) Carbon Analysis

Carbon content (OC, EC) in the PM_10_ fraction was determined by the thermo-optical transmittance method (TOT). Analyses were conducted on a Carbon Aerosol Analyzer (Sunset Laboratory Inc., Amsterdam, The Netherlands) with a flame ionization detector, FID, [112,146,147] using the Quartz (NIOSH-like) protocol. To ensure QA/QC and to prove the consistent operation of the instrument, the inner standard, an external sucrose aqueous solution, and a cross-method procedure were used. The results of the recovery are (100 ± 4) % with a relative standard deviation of RSD < 5% [112,115,148].

##### Water-Soluble-Anions Analysis

Water-soluble acidic anions (Cl^−^, NO_3_^−^, SO_4_^2−^) were analysed by ion chromatography using a Dionex DX 120 chromatograph equipped with suppressed conductivity detection (Dionex, Sunnyvale, CA, USA), Dionex AS14: 4 mm Analytical Column + AG14: 4 mm Guard Column. The eluent was 3.5 mM Na_2_CO_3_/1 mM NaHCO_3_ solution. Prior to the analysis, filters were extracted by ultrasound in 18 Milli-Q ultrapure water (resistivity ≥ 18.3 MΩcm). For the instrument calibration and quantitative determination of each component, a commercial standard-mixture solution of anions (Dionex) was used [109,110]. The limits of detection (LOD) for each measured analyte are presented in the Supplementary Materials (Supplementary Table S3).

### 4.5. Data Processing

Air pollution exposure data obtained from January 2011 to January 2015 in the form of 24 h mass concentrations of pollutants were subjected to factor analysis. As the distributions of all the measurements were right-skewed, the factor analyses were performed on the log(10) transformations of the measurements. Nickel was excluded from this factor analysis owing to a high number of measurements below the detection limit. To avoid the potential bias related to partially missing measurements, we applied multiple imputations (n = 50) based on the correlation structure and replaced the missing measurements with the mean of the imputed variables, leading to 1492 complete daily measurements. The factor analysis included log PM_10_, PM_2.5_, PM_1_, as well as the above-cited metals (Pb, Mn, Cd, As, Cu, Fe, Zn) and other pollutants EC, OC, SO_4_^2−^, NO^3−^, Cl^−^. B[a]P. All pollutants were measured in the PM_10_ fraction and standardized by PM_10_. The factor analysis thus included 16 variables. The number of factors retained was based on a criterion of an eigenvalue greater than 1. A post hoc varimax rotation was then performed to select the factors close to the original variables. Four factors were retained: a PM factor, 2 metal factors (F2–Mn, Cu, Fe, Zn, and F4–Pb, Cd, As, Zn), and the last F3 factor correlated to NO_3_^−^, Cl^−^, OC, EC and B[a]P—negatively with SO_4_^2−^.

The average air pollution exposure was calculated for each participant. Finally, predicted factor-specific indices for all subjects were generated for three time windows (last 3, 7, and 30 days) based on the prediction equation obtained using all the exposure measurements.

### 4.6. Statistical Analysis

When comparing the exposure measurements according to the time windows, we applied a standard simple analysis of variance. For the substance, for which some measurements were below the limit of quantification, this was replaced by interval regression followed by a Wald test [149,150].

The regression models used to assess the effect of air pollution on the CBMN assay parameters were either the linear mixed model for NDI or mixed Poisson models for count data (total number of MNi, NPBs, or NBUDs). The random-effect parameter (the measurement date) was included to account for the correlation induced by the fact that all blood cells collected on the same day were assigned the same exposure measurements. For each parameter, the first step consisted of a stepwise regression, including all potential confounding variables. All the confounders that were even marginally significant were included in the models in which the effect of the environmental exposure was tested. As (except for B[a]P) no a priori hypothesis of effect had been formulated, the effect of air pollution was assessed using the computed factor-specific indices. For each parameter and time window, three models were fitted—all including the parameter-specific confounding factors. The first model included only factor 1, which assessed the global effect of PM. The second model included the PM factor F1 and the two metal factors (F2 and F4), thus assessing a possible metal-specific effect within the PM. The third model included the PM factor F1, and the last factor, F3, correlated with the other substances. A final series of models included the unstandardized exposure to B[a]P, as this is a recognized carcinogen. 

## Figures and Tables

**Table 1:** Population characteristics and lifestyle factors of the study population (mean values ± standard deviation of the mean).

	Women	Men	Total
***N***	89	41	130
**Age (years)**	38.06 ± 13.94	38.12 ± 11.99	38.08 ± 13.31
**Age range (years)**	19–80	24–68	19–80
**BMI (kg/m^2^)**	22.99 ± 3.92	25.74 ± 3.00	23.99 ± 3.84
**Current smokers**	27	11	38
**Alcohol consumers**	51	32	83
**Physical activity**	26	24	50
**Family history of cancer**	39	11	50

*N*, number of subjects; BMI, body mass index.

**Table 2:** Mean values ± standard deviation of the cytokinesis-block micronucleus (CBMN) assay parameters (total number of micronuclei, nucleoplasmic bridges, nuclear buds per 1000 cells, and nuclear division index) in peripheral blood lymphocytes of the general population.

	Women	Men	Total
**MNi**			
Mean	5.28 ± 2.81	4.76 ± 2.84	5.12 ± 2.82
Range	1–13	1–12	1–13
**NPBs**			
Mean	1.09 ± 1.35	1.51 ± 1.79	1.22 ± 1.51
Range	0–7	0–7	0–7
**NBUDs**			
Mean	3.43 ± 2.05	3.51 ± 2.21	3.45 ± 2.10
Range	0–10	0–10	0–10
**NDI**			
Mean	2.01 ± 0.12	1.98 ± 0.12	2.00 ± 0.12
Range	1.60–2.38	1.61–2.16	1.60–2.38

MNi, micronuclei; NPBs, nucleoplasmic bridges; NBUDs, nuclear buds; NDI, nuclear division index.

**Table 3:** Air pollution exposure of participants during different time windows (3, 7, and 30 days before blood sampling).

Pollutant	Average 3 Days Before	Average 7 Days Before	Average 30 Days Before
**PM_10_ (µg/m^3^)**			
Mean ± SD	29 ± 15	30 ± 13	32 ± 13
Range	11–81	15–67	13–60
**PM_2.5_ (µg/m^3^)**			
Mean ± SD	23 ± 14	23 ± 13	25 ± 12
Range	9–68	9–59	8–50
**PM_1_ (µg/m^3^)**			
Mean ± SD	16 ± 9	17 ± 9	18 ± 8
Range	7–39	7–50	7–35
OC (µg/m^3^) *			
Mean ± SD	8.16 ± 4.42	8.33 ± 4.37	8.74 ± 4.28
Range	3.02–25.40	3.50–19.48	3.74–16.59
EC (µg/m^3^) *			
Mean ± SD	1.07 ± 0.57	1.14 ± 0.51	1.10 ± 0.39
Range	0.22–2.94	0.27–2.36	0.42–1.84
SO_4_^2−^ (µg/m^3^) *			
Mean ± SD	4.31 ± 5.22	4.09 ± 3.60	4.40 ± 2.28
Range	0.51–31.30	1.40–19.88	1.32–10.64
NO_3_^−^ (µg/m^3^) *			
Mean ± SD	3.17 ± 3.31	3.41 ± 3.22	3.58 ± 2.90
Range	0.25–13.10	0.34–16.10	0.34–11.54
Cl^−^ (µg/m^3^) *			
Mean ± SD	0.20 ± 0.29	0.22 ± 0.31	0.21 ± 0.24
Range	0.01–1.42	0.01–1.44	0.01–0.97
Pb (µg/m^3^) *			
Mean ± SD	0.006 ± 0.005	0.006 ± 0.003	0.006 ± 0.003
Range	0.002–0.036	0.003–0.021	0.003–0.013
Mn (µg/m^3^) *			
Mean ± SD	0.005 ± 0.002	0.006 ± 0.002	0.005 ± 0.001
Range	0.002–0.012	0.003–0.010	0.003–0.011
Cd (ng/m^3^) *			
Mean ± SD	0.217 ± 0.161	0.222 ± 0.161	0.229 ± 0.128
Range	0.068–0.884	0.068–0.837	0.072–0.666
As (ng/m^3^) *			
Mean ± SD	0.554 ± 0.491	0.569 ± 0.461	0.562 ± 0.220
Range	0.156–2.310	0.231–2.852	0.269–1.191
Ni (ng/m^3^) *			
Mean ± SD	0.977 ± 1.052	1.057 ± 0.956	1.169 ± 1.078
Range	LOD-5.018	LOD-5.454	0.067–6.240
Cu (µg/m^3^) *			
Mean ± SD	0.012 ± 0.005	0.013 ± 0.005	0.013 ± 0.004
Range	0.004–0.034	0.005–0.025	0.006–0.033
Fe (µg/m^3^) *			
Mean ± SD	0.300 ± 0.153	0.325 ± 0.121	0.303 ± 0.078
Range	0.086–0.958	0.142–0.712	0.179–0.612
Zn (µg/m^3^) *			
Mean ± SD	0.021 ± 0.010	0.021 ± 0.009	0.022 ± 0.007
Range	0.008–0.057	0.011–0.055	0.011–0.044
B[a]P (ng/m^3^) *			
Mean ± SD	1.033 ± 1.242	1.024 ± 1.075	1.151 ± 1.081
Range	LOD-5.307	0.039–3.517	0.050–3.508

* In PM_10_ particle fraction. SD, standard deviation; LOD, limit of detection; PM, particulate matter; OC, organic carbon; EC, elemental carbon; B[a]P, benzo(a)pyrene.

## Data Availability

The original contributions generated for this study are included in the article. Further inquiries can be directed to the corresponding author upon reasonable request.

## Supplementary Materials

The supporting information can be downloaded at: https://www.mdpi.com/article/10.3390/ijms231710083/s1.

## References

[1] WHO (2018). WHO Global Urban Ambient Air Pollution Database.

[2] Lelieveld J., Pöschl U. (2017). Chemists can help to solve the air-pollution health crisis. Nature.

[3] Lelieveld J., Pozzer A., Pöschl U., Fnais M., Haines A., Münzel T. (2020). Loss of life expectancy from air pollution compared to other risk factors: A worldwide perspective. Cardiovasc. Res..

[4] Boogaard H., Walker K., Cohen A.J. (2019). Air pollution: The emergence of a major global health risk factor. Int. Health.

[5] Khomenko S., Cirach M., Pereira-Barboza E., Mueller N., Barrera-Gómez J., Rojas-Rueda D., de Hoogh K., Hoek G., Nieuwenhuijsen M. (2021). Premature mortality due to air pollution in European cities: A health impact assessment. Lancet Planet. Health.

[6] EEA (2021). Air Quality in Europe—2021 Report—European Environment Agency (EEA).

[7] European Commission (2008). Directive 2008/50/EC of the European Parliament and of the Council of 21 May 2008 on Ambient Air Quality and Cleaner Air for Europe. Off. J. Eur. Commun..

[8] European Commission (2005). Directive 2004/107/EC of the European Parliament and of the Council of 15/12/2004 Relating to Arsenic, Cadmium, Mercury, Nickel and Polycyclic Aromatic Hydrocarbons in Ambient Air. Off. J. Eur. Union.

[9] WHO (2006). Air Quality Guidelines: Global Update 2005: Particulate Matter, Ozone, Nitrogen Dioxide, and Sulfur Dioxide.

[10] Stanek L.W., Brown J.S., Stanek J., Gift J., Costa D.L. (2011). Air Pollution Toxicology—A Brief Review of the Role of the Science in Shaping the Current Understanding of Air Pollution Health Risks. Toxicol. Sci..

[11] EEA (2017). Air Quality in Europe—2017 Report.

[12] Jaafari J., Naddafi K., Yunesian M., Nabizadeh R., Hassanvand M.S., Ghozikali M.G., Shamsollahi H.R., Nazmara S., Yaghmaeian K. (2020). Characterization, risk assessment and potential source identification of PM_10_ in Tehran. Microchem. J..

[13] Agudelo-Castañeda D.M., Teixeira E.C., Schneider I.L., Lara S.R., Silva L.F.O. (2017). Exposure to polycyclic aromatic hydrocarbons in atmospheric PM_1.0_ of urban environments: Carcinogenic and mutagenic respiratory health risk by age groups. Environ. Pollut..

[14] Bhardawaj A., Habib G., Nema A.K., Kumar A., Singh S. (2017). A Review of Ultrafine Particle-Related Pollution during Vehicular Motion, Health Effects and Control. J. Environ. Sci. Public Health.

[15] Dandotiya B. (2019). Health Effects of Air Pollution in Urban Environment. Climate Change and Its Impact on Ecosystem Services and Biodiversity in Arid and Semi-Arid Zones.

[16] De Marco A., Proietti C., Anav A., Ciancarella L., D’Elia I., Fares S., Fornasier M.F., Fusaro L., Gualtieri M., Manes F. (2019). Impacts of air pollution on human and ecosystem health, and implications for the National Emission Ceilings Directive: Insights from Italy. Environ. Int..

[17] Habre R., Zhou H., Eckel S.P., Enebish T., Fruin S., Bastain T., Rappaport E., Gilliland F. (2018). Short-term effects of airport-associated ultrafine particle exposure on lung function and inflammation in adults with asthma. Environ. Int..

[18] Idowu O., Semple K., Ramadass K., O’Connor W., Hansbro P., Thavamani P. (2019). Beyond the obvious: Environmental health implications of polar polycyclic aromatic hydrocarbons. Environ. Int..

[19] Khamraev K., Cheriyan D., Choi J.-H. (2020). A review on health risk assessment of PM in the construction industry—Current situation and future directions. Sci. Total Environ..

[20] Rovira J., Domingo J.L., Schuhmacher M. (2020). Air quality, health impacts and burden of disease due to air pollution (PM_10_, PM_2.5_, NO_2_ and O_3_): Application of AirQ+ model to the Camp de Tarragona County (Catalonia, Spain). Sci. Total Environ..

[21] Samoli E., Stafoggia M., Rodopoulou S., Ostro B., Alessandrini E., Basagaña X., Díaz J., Faustini A., Gandini M., Karanasiou A. (2014). Which specific causes of death are associated with short term exposure to fine and coarse particles in Southern Europe? Results from the MED-PARTICLES project. Environ. Int..

[22] Segersson D., Eneroth K., Gidhagen L., Johansson C., Omstedt G., Nylén A.E., Forsberg B. (2017). Health Impact of PM_10_, PM_2.5_ and Black Carbon Exposure Due to Different Source Sectors in Stockholm, Gothenburg and Umea, Sweden. Int. J. Environ. Res. Public Health.

[23] Tian M., Liang B., Zhang L., Hu H., Yang F., Peng C., Chen Y., Jiang C., Wang J. (2021). Measurement of size-segregated airborne particulate bound polycyclic aromatic compounds and assessment of their human health impacts—A case study in a megacity of southwest China. Chemosphere.

[24] Liu D., Lin T., Syed J.H., Cheng Z., Xu Y., Li K., Zhang G., Li J. (2017). Concentration, source identification, and exposure risk assessment of PM_2.5_-bound parent PAHs and nitro-PAHs in atmosphere from typical Chinese cities. Sci. Rep..

[25] Li J., Chen X., Wang Z., Du H., Yang W., Sun Y., Hu B., Li J., Wang W., Wang T. (2018). Radiative and heterogeneous chemical effects of aerosols on ozone and inorganic aerosols over East Asia. Sci. Total Environ..

[26] Li Q., Wang Y.-Y., Guo Y., Zhou H., Wang X., Wang Q., Shen H., Zhang Y., Yan D., Zhang Y. (2018). Effect of airborne particulate matter of 2.5 μm or less on preterm birth: A national birth cohort study in China. Environ. Int..

[27] Brauer M., Freedman G., Frostad J., Van Donkelaar A., Martin R., Dentener F., Van Dingenen R., Estep K., Amini H., Apte J. (2016). Ambient Air Pollution Exposure Estimation for the Global Burden of Disease 2013. Environ. Sci. Technol..

[28] Knudsen L.E., Andersen Z.J., Sram R.J., Kohlová M.B., Gurzau E.S., Fucic A., Gribaldo L., Rossner P., Rossnerova A., Máca V. (2017). Perinatal health in the Danube region—New birth cohort justified. Rev. Environ. Health.

[29] Santovito A., Gendusa C., Cervella P., Traversi D. (2020). In vitro genomic damage induced by urban fine particulate matter on human lymphocytes. Sci. Rep..

[30] Anderson J.O., Thundiyil J.G., Stolbach A. (2012). Clearing the Air: A Review of the Effects of Particulate Matter Air Pollution on Human Health. J. Med. Toxicol..

[31] Beelen R., Raaschou-Nielsen O., Stafoggia M., Andersen Z.J., Weinmayr G., Hoffmann B., Wolf K., Samoli E., Fischer P., Nieuwenhuijsen M. (2014). Effects of long-term exposure to air pollution on natural-cause mortality: An analysis of 22 European cohorts within the multicentre ESCAPE project. Lancet.

[32] Cesaroni G., Forastiere F., Stafoggia M., Andersen Z.J., Badaloni C., Beelen R., Caracciolo B., De Faire U., Erbel R., Eriksen K.T. (2014). Long term exposure to ambient air pollution and incidence of acute coronary events: Prospective cohort study and meta-analysis in 11 European cohorts from the ESCAPE Project. BMJ.

[33] Landrigan P.J., Fuller R., Acosta N.J.R., Adeyi O., Arnold R., Basu N., Baldé A.B., Bertollini R., Bose-O’Reilly S., Boufford J.I. (2018). The Lancet Commission on pollution and health. Lancet.

[34] Lelieveld J., Evans J.S., Fnais M., Giannadaki D., Pozzer A. (2015). The contribution of outdoor air pollution sources to premature mortality on a global scale. Nature.

[35] Myers S.S. (2017). Planetary health: Protecting human health on a rapidly changing planet. Lancet.

[36] Raaschou-Nielsen O., Andersen Z.J., Beelen R., Samoli E., Stafoggia M., Weinmayr G., Hoffmann B., Fischer P., Nieuwenhuijsen M.J., Brunekreef B. (2013). Air pollution and lung cancer incidence in 17 European cohorts: Prospective analyses from the European Study of Cohorts for Air Pollution Effects (ESCAPE). Lancet Oncol..

[37] Shah A.S.V., Langrish J.P., Nair H., McAllister D.A., Hunter A.L., Donaldson K., Newby D.E., Mills N.L. (2013). Global association of air pollution and heart failure: A systematic review and meta-analysis. Lancet.

[38] Žero S., Žužul S., Huremović J., Pehnec G., Bešlić I., Rinkovec J., Godec R., Kittner N., Pavlović K., Požar N. (2022). New Insight into the Measurements of Particle-Bound Metals in the Urban and Remote Atmospheres of the Sarajevo Canton and Modeled Impacts of Particulate Air Pollution in Bosnia and Herzegovina. Environ. Sci. Technol..

[39] WHO (2013). Health Effects of Particulate Matter, Policy Implications for Countries in Eastern Europe, Caucasus and Central Asia.

[40] WHO (2013). Review of Evidence on Health Aspects of Air Pollution—REVIHAAP Project: Final Technical Report.

[41] Eze I., Hemkens L., Bucher H.C., Hoffmann B., Schindler C., Künzli N., Schikowski T., Probst-Hensch N.M. (2015). Association between Ambient Air Pollution and Diabetes Mellitus in Europe and North America: Systematic Review and Meta-Analysis. Environ. Health Perspect..

[42] Lewtas J. (2007). Air pollution combustion emissions: Characterization of causative agents and mechanisms associated with cancer, reproductive, and cardiovascular effects. Mutat. Res. Mutat. Res..

[43] Peixoto M.S., Galvão M.F.D.O., de Medeiros S.R.B. (2017). Cell death pathways of particulate matter toxicity. Chemosphere.

[44] Rao X., Patel P., Puett R., Rajagopalan S. (2015). Air Pollution as a Risk Factor for Type 2 Diabetes. Toxicol. Sci..

[45] Daellenbach K.R., Uzu G., Jiang J., Cassagnes L.-E., Leni Z., Vlachou A., Stefenelli G., Canonaco F., Weber S., Segers A. (2020). Sources of particulate-matter air pollution and its oxidative potential in Europe. Nature.

[46] Weber R. (2020). Potentially harmful aerosols concentrate in European urban centres. Nature.

[47] Fenech M. (2007). Cytokinesis-block micronucleus cytome assay. Nat. Protoc..

[48] Gajski G., Gerić M., Oreščanin V., Garaj-Vrhovac V. (2018). Cytokinesis-block micronucleus cytome assay parameters in peripheral blood lymphocytes of the general population: Contribution of age, sex, seasonal variations and lifestyle factors. Ecotoxicol. Environ. Saf..

[49] Sommer S., Buraczewska I., Kruszewski M. (2020). Micronucleus Assay: The State of Art, and Future Directions. Int. J. Mol. Sci..

[50] Kopjar N., Kašuba V., Milić M., Rozgaj R., Želježić D., Gajski G., Mladinic M., Garaj-Vrhovac V. (2010). Normal and Cut-Off Values of the Cytokinesis-Block Micronucleus Assay on Peripheral Blood Lymphocytes in the Croatian General Population. Arch. Ind. Hyg. Toxicol..

[51] Hayashi M. (2016). The micronucleus test—Most widely used in vivo genotoxicity test. Genes Environ..

[52] Garaj-Vrhovac V., Gajski G., Ravlić S. (2008). Efficacy of HUMN criteria for scoring the micronucleus assay in human lymphocytes exposed to a low concentration of p,p’-DDT. Braz. J. Med. Biol. Res..

[53] Gajski G., Gerić M., Oreščanin V., Garaj-Vrhovac V. (2013). Cytogenetic status of healthy children assessed with the alkaline comet assay and the cytokinesis-block micronucleus cytome assay. Mutat. Res. Toxicol. Environ. Mutagen..

[54] Gajski G., Gerić M., Lovrenčić M.V., Božičević S., Rubelj I., Nanić L., Vidaček N., Bendix L., Peraica M., Rašić D. (2018). Analysis of health-related biomarkers between vegetarians and non-vegetarians: A multi-biomarker approach. J. Funct. Foods.

[55] Gerić M., Popić J., Gajski G., Garaj-Vrhovac V. (2019). Cytogenetic status of interventional radiology unit workers occupationally exposed to low-dose ionising radiation: A pilot study. Mutat. Res. Toxicol. Environ. Mutagen..

[56] Rossnerova A., Spatova M., Rossner P., Solansky I., Sram R.J. (2009). The impact of air pollution on the levels of micronuclei measured by automated image analysis. Mutat. Res. Mol. Mech. Mutagen..

[57] Bolognesi C., Merlo F., Rabboni R., Valerio F., Abbondandolo A. (1997). Cytogenetic biomonitoring in traffic police workers: Micronucleus test in peripheral blood lymphocytes. Environ. Mol. Mutagen..

[58] Fenech M., Chang W.P., Kirsch-Volders M., Holland N., Bonassi S., Zeiger E. (2003). HUman MicronNucleus project HUMN Project: Detailed Description of the Scoring Criteria for the Cytokinesis-Block Micronucleus Assay Using Isolated Human Lymphocyte Cultures. Mutat. Res..

[59] Fenech M. (2020). Cytokinesis-Block Micronucleus Cytome Assay Evolution into a More Comprehensive Method to Measure Chromosomal Instability. Genes.

[60] Gekara N.O. (2017). DNA damage-induced immune response: Micronuclei provide key platform. J. Cell Biol..

[61] Thomas P., Umegaki K., Fenech M. (2003). Nucleoplasmic bridges are a sensitive measure of chromosome rearrangement in the cytokinesis-block micronucleus assay. Mutagenesis.

[62] Bonassi S., Znaor A., Ceppi M., Lando C., Chang W.P., Holland N., Kirsch-Volders M., Zeiger E., Ban S., Barale R. (2007). An increased micronucleus frequency in peripheral blood lymphocytes predicts the risk of cancer in humans. Carcinogenesis.

[63] Bonassi S., El-Zein R., Bolognesi C., Fenech M. (2011). Micronuclei frequency in peripheral blood lymphocytes and cancer risk: Evidence from human studies. Mutagenesis.

[64] Nersesyan A., Mišík M., Cherkas A., Serhiyenko V., Staudinger M., Holota S., Yatskevych O., Melnyk S., Holzmann K., Knasmüller S. (2021). Use of Micronucleus Experiments for the Detection of Human Cancer Risks: A Brief Overview. Proc. Shevchenko Sci. Soc. Med. Sci..

[65] Faust F., Kassie F., Knasmüller S., Kevekordes S., Mersch-Sundermann V. (2004). Use of primary blood cells for the assessment of exposure to occupational genotoxicants in human biomonitoring studies. Toxicology.

[66] Faust F., Kassie F., Knasmüller S., Boedecker R.H., Mann M., Mersch-Sundermann V. (2004). The use of the alkaline comet assay with lymphocytes in human biomonitoring studies. Mutat. Res. Mutat. Res..

[67] Gajski G., Gerić M., Marques de Oliveira M.M., Barreira Morais S., Martins Rodrigues F.P.L. (2022). Cytogenetic Methods for Measuring Effects of Occupational Exposure. An Essential Guide to Occupational Exposure.

[68] Pöschl U. (2005). Atmospheric Aerosols: Composition, Transformation, Climate and Health Effects. Angew. Chem. Int. Ed..

[69] Van Dingenen R., Raes F., Putaud J.-P., Baltensperger U., Charron A., Facchini M.-C., Decesari S., Fuzzi S., Gehrig R., Hansson H.-C. (2004). A European aerosol phenomenology—1: Physical characteristics of particulate matter at kerbside, urban, rural and background sites in Europe. Atmos. Environ..

[70] Putaud J.-P., Van Dingenen R., Alastuey A., Bauer H., Birmili W., Cyrys J., Flentje H., Fuzzi S., Gehrig R., Hansson H.C. (2010). A European aerosol phenomenology—3: Physical and chemical characteristics of particulate matter from 60 rural, urban, and kerbside sites across Europe. Atmos. Environ..

[71] Cavalli F., Alastuey A., Areskoug H., Ceburnis D., Čech J., Genberg J., Harrison R.M., Jaffrezo J.L., Kiss G., Laj P. (2016). A European aerosol phenomenology-4: Harmonized concentrations of carbonaceous aerosol at 10 regional background sites across Europe. Atmos. Environ..

[72] Zanatta M., Gysel M., Bukowiecki N., Müller T., Weingartner E., Areskoug H., Fiebig M., Yttri K.E., Mihalopoulos N., Kouvarakis G. (2016). A European aerosol phenomenology-5: Climatology of black carbon optical properties at 9 regional background sites across Europe. Atmos. Environ..

[73] Pandolfi M., Alados-Arboledas L., Alastuey A., Andrade M., Angelov C., Artiñano B., Backman J., Baltensperger U., Bonasoni P., Bukowiecki N. (2018). A European aerosol phenomenology—6: Scattering properties of atmospheric aerosol particles from 28 ACTRIS sites. Atmos. Chem. Phys..

[74] Bressi M., Cavalli F., Putaud J.P., Fröhlich R., Petit J.E., Aas W., Äijälä M., Alastuey A., Allan J.D., Aurela M. (2021). A European aerosol phenomenology—7: High-time resolution chemical characteristics of submicron particulate matter across Europe. Atmos. Environ. X.

[75] Putaud J.-P., Raes F., Van Dingenen R., Brüggemann E., Facchini M.-C., Decesari S., Fuzzi S., Gehrig R., Hüglin C., Laj P. (2004). A European aerosol phenomenology—2: Chemical characteristics of particulate matter at kerbside, urban, rural and background sites in Europe. Atmos. Environ..

[76] Angerer J., Bird M.G., Burke T.A., Doerrer N.G., Needham L., Robison S.H., Sheldon L., Zenick H. (2006). Strategic Biomonitoring Initiatives: Moving the Science Forward. Toxicol. Sci..

[77] Angerer J., Ewers U., Wilhelm M. (2007). Human biomonitoring: State of the art. Int. J. Hyg. Environ. Health.

[78] WHO (2015). Human Biomonitoring: Facts and Figures.

[79] Ladeira C., Viegas S. (2016). Human Biomonitoring—An overview on biomarkers and their application in Occupational and Environmental Health. Biomonitoring.

[80] Lionetto M.G., Caricato R., Giordano M.E. (2019). Pollution Biomarkers in Environmental and Human Biomonitoring. Open Biomarkers J..

[81] Bajpayee M., Dhawan A. (2014). Biomarkers for Monitoring Adverse Health Effects of Air Pollution in Humans. J. Transl. Toxicol..

[82] Jeddi M.Z., Hopf N.B., Viegas S., Price A.B., Paini A., van Thriel C., Benfenati E., Ndaw S., Bessems J., Behnisch P.A. (2021). Towards a systematic use of effect biomarkers in population and occupational biomonitoring. Environ. Int..

[83] Gajski G., Gerić M., Semren T., Lovaković B.T., Oreščanin V., Pizent A. (2020). Application of the comet assay for the evaluation of DNA damage from frozen human whole blood samples: Implications for human biomonitoring. Toxicol. Lett..

[84] JakovljeviĆ I., Pehnec G., Sisovic A., Vađić V., Davila S., Godec R. (2016). Concentrations of PAHs and other gaseous pollutants in the atmosphere of a rural area. J. Environ. Sci. Health Part A.

[85] Godec R., Jakovljević I., Šega K., Čačković M., Bešlić I., Davila S., Pehnec G. (2016). Carbon species in PM_10_ particle fraction at different monitoring sites. Environ. Pollut..

[86] Jakovljević I., Pehnec G., Vađić V., Čačković M., Tomašić V., Jelinić J.D. (2018). Polycyclic Aromatic Hydrocarbons in PM_10_, PM_2.5_ and PM_1_ Particle Fractions in an Urban Area. Air Qual. Atmos. Health.

[87] JakovljeviĆ I., Pehnec G., Vadjić V., Šišović A., Davila S., Bešlić I. (2015). Carcinogenic activity of polycyclic aromatic hydrocarbons bounded on particle fraction. Environ. Sci. Pollut. Res..

[88] Šišović A., Pehnec G., Jakovljević I., Hujić M., Vađić V., Bešlić I. (2012). Polycyclic Aromatic Hydrocarbons at Different Crossroads in Zagreb, Croatia. Bull. Environ. Contam. Toxicol..

[89] Pehnec G., Jakovljević I., Šišović A., Bešlić I., Vađić V. (2016). Influence of ozone and meteorological parameters on levels of polycyclic aromatic hydrocarbons in the air. Atmos. Environ..

[90] Callén M.S., Lopez J.M., Mastral A.M. (2010). Seasonal variation of benzo(a)pyrene in the Spanish airborne PM_10_. Multivariate linear regression model applied to estimate BaP concentrations. J. Hazard. Mater..

[91] Caricchia A.M., Chiavarini S., Pezza M. (1999). Polycyclic aromatic hydrocarbons in the urban atmospheric particulate matter in the city of Naples (Italy). Atmos. Environ..

[92] Pehnec G., Jakovljević I., Godec R., Štrukil Z.S., Žero S., Huremović J., Džepina K. (2020). Carcinogenic organic content of particulate matter at urban locations with different pollution sources. Sci. Total Environ..

[93] Bari M.A., Baumbach G., Kuch B., Scheffknecht G. (2010). Particle-phase concentrations of polycyclic aromatic hydrocarbons in ambient air of rural residential areas in southern Germany. Air Qual. Atmos. Health.

[94] (2020). Official Gazette No. 77 Uredba o Razinama Onečišćujućih Tvari u Zraku/Regulation on Levels of Air Pollutants. Croatia.

[95] Vađić V., Žužul S., Rinkovec J., Pehnec G. (2013). Metali u Sitnim Česticama u Zraku Zagreba. Sigurnost.

[96] Bešlić I., Burger J., Cadoni F., Centioli D., Kranjc I., Van den Bril B., Rinkovec J., Šega K., Zang T., Žužul S. (2020). Determination of As, Cd, Ni and Pb in PM_10_—Comparison of Different Sample Work-up and Analysis Methods. Gefahrst. Reinhalt. Der Luft.

[97] Vadjić V., Žužul S., Pehnec G. (2010). Zinc Levels in Suspended Particulate Matter in Zagreb Air. Bull. Environ. Contam. Toxicol..

[98] Joksic J., Jovasevic-Stojanovic M., Bartonova A., Radenkovic M., Yttri K.-E., Matic-Besarabic S., Ignjatovic L. (2009). Physical and chemical characterization of the particulate matter suspended in aerosols from the urban area of Belgrade. J. Serbian Chem. Soc..

[99] Perišić M., Rajšić S., Šoštarić A., Mijić Z., Stojić A. (2017). Levels of PM_10_-bound species in Belgrade, Serbia: Spatio-temporal distributions and related human health risk estimation. Air Qual. Atmos. Health.

[100] Žero S., Huremović J., Memić M., Muhić-Šarac T. (2017). Determination of total and bioaccessible metals in airborne particulate matter from an urban and a rural area at Sarajevo. Toxicol. Environ. Chem..

[101] Aksu A. (2015). Sources of metal pollution in the urban atmosphere (A case study: Tuzla, Istanbul). J. Environ. Health Sci. Eng..

[102] Hernández-Pellón A., Fernández-Olmo I. (2019). Airborne concentration and deposition of trace metals and metalloids in an urban area downwind of a manganese alloy plant. Atmos. Pollut. Res..

[103] Albuquerque M., Coutinho M., Rodrigues J., Ginja J., Borrego C. (2017). Long-term monitoring of trace metals in PM_10_ and total gaseous mercury in the atmosphere of Porto, Portugal. Atmos. Pollut. Res..

[104] Contini D., Cesari D., Donateo A., Chirizzi D., Belosi F. (2014). Characterization of PM_10_ and PM_2.5_ and Their Metals Content in Different Typologies of Sites in South-Eastern Italy. Atmosphere.

[105] (2005). Official Gazette No. 133/2005 Uredba o Graničnim Vrijednostima Onečišćujućih Tvari u Zraku/Regulation on Levels of Air Pollutants. Croatia.

[106] Tunno B.J., Shmool J.L.C., Michanowicz D.R., Tripathy S., Chubb L., Kinnee E., Cambal L., Roper C., Clougherty J.E. (2016). Spatial variation in diesel-related elemental and organic PM_2.5_ components during workweek hours across a downtown core. Sci. Total Environ..

[107] Rivas I., Viana M., Moreno T., Bouso L., Pandolfi M., Alvarez-Pedrerol M., Forns J., Alastuey A., Sunyer J., Querol X. (2015). Outdoor infiltration and indoor contribution of UFP and BC, OC, secondary inorganic ions and metals in PM_2.5_ in schools. Atmos. Environ..

[108] Perrone M.G., Vratolis S., Georgieva E., Török S., Šega K., Veleva B., Osán J., Bešlić I., Kertész Z., Pernigotti D. (2018). Sources and geographic origin of particulate matter in urban areas of the Danube macro-region: The cases of Zagreb (Croatia), Budapest (Hungary) and Sofia (Bulgaria). Sci. Total Environ..

[109] Čačković M., Šega K., Vađić V., Bešlić I. (2008). Characterisation of Major Acidic Anions in TSP and PM_10_ in Zagreb Air. Bull. Environ. Contam. Toxicol..

[110] Čačković M., Vađić V., Šega K., Beslic I. (2009). Acidic Anions in PM_10_ Particle Fraction in Zagreb Air, Croatia. Bull. Environ. Contam. Toxicol..

[111] Godec R., Jakovljević I., Davila S., Šega K., Bešlić I., Rinkovec J., Pehnec G. (2021). Air pollution levels near crossroads with different traffic density and the estimation of health risk. Environ. Geochem. Health.

[112] Godec R., Čačković M., Šega K., Bešlić I. (2012). Winter Mass Concentrations of Carbon Species in PM_10_, PM_2.5_ and PM_1_ in Zagreb Air, Croatia. Bull. Environ. Contam. Toxicol..

[113] Alves C.A., Evtyugina M., Vicente A.M.P., Vicente E.D., Nunes T.V., Silva P.M.A., Duarte M.A.C., Pio C.A., Amato F., Querol X. (2018). Chemical profiling of PM_10_ from urban road dust. Sci. Total Environ..

[114] Jeong C.-H., Wang J.M., Hilker N., Debosz J., Sofowote U., Su Y., Noble M., Healy R.M., Munoz T., Dabek-Zlotorzynska E. (2019). Temporal and spatial variability of traffic-related PM_2.5_ sources: Comparison of exhaust and non-exhaust emissions. Atmos. Environ..

[115] Godec R., Šega K., Bešlić I., Davila S., Ovašević-Stojanović M., Bartoňová A. (2016). Carbon Mass Concentrations in Southern Zagreb during a Five-Year Period. Proceedings of the 5th WeBIOPATR Workshop & Conference.

[116] Jiang N., Duan S.G., Yu X., Zhang R.Q., Wang K. (2018). Comparative major components and health risks of toxic elements and polycyclic aromatic hydrocarbons of PM_2.5_ in winter and summer in Zhengzhou: Based on three-year data. Atmos. Res..

[117] Tao J., Shen Z., Zhu C., Yue J., Cao J., Liu S., Zhu L., Zhang R. (2012). Seasonal variations and chemical characteristics of sub-micrometer particles (PM_1_) in Guangzhou, China. Atmos. Res..

[118] Nielsen T., Jørgensen H.E., Larsen J.C., Poulsen M. (1996). City air pollution of polycyclic aromatic hydrocarbons and other mutagens: Occurrence, sources and health effects. Sci. Total Environ..

[119] Ravindra K., Sokhi R., Van Grieken R. (2008). Atmospheric polycyclic aromatic hydrocarbons: Source attribution, emission factors and regulation. Atmos. Environ..

[120] Kim K.-H., Jahan S.A., Kabir E., Brown R.J.C. (2013). A review of airborne polycyclic aromatic hydrocarbons (PAHs) and their human health effects. Environ. Int..

[121] Takamura-Enya T., Suzuki H., Hisamatsu Y. (2006). Mutagenic activities and physicochemical properties of selected nitrobenzanthrones. Mutagenesis.

[122] Marquis O., Miaud C., Ficetola G.F., Bocher A., Mouchet F., Guittonneau S., Devaux A. (2009). Variation in genotoxic stress tolerance among frog populations exposed to UV and pollutant gradients. Aquat. Toxicol..

[123] Toyooka T., Ohnuki G., Ibuki Y. (2008). Solar-simulated light-exposed benzo[a]pyrene induces phosphorylation of histone H2AX. Mutat. Res. Toxicol. Environ. Mutagen..

[124] Arfsten D.P., Schaeffer D., Mulveny D.C. (1996). The Effects of Near Ultraviolet Radiation on the Toxic Effects of Polycyclic Aromatic Hydrocarbons in Animals and Plants: A Review. Ecotoxicol. Environ. Saf..

[125] Yu H., Xia Q., Yan J., Herreno-Saenz D., Wu Y.-S., Tang I.-W., Fu P.P. (2006). Photoirradiation of Polycyclic Aromatic Hydrocarbons with UVA Light—A Pathway Leading to the Generation of Reactive Oxygen Species, Lipid Peroxidation, and DNA Damage. Int. J. Environ. Res. Public Health.

[126] Bukowska B., Mokra K., Michałowicz J. (2022). Benzo[a]pyrene—Environmental Occurrence, Human Exposure, and Mechanisms of Toxicity. Int. J. Mol. Sci..

[127] O’Callaghan-Gordo C., Fthenou E., Pedersen M., Espinosa A., Chatzi L., Beelen R., Chalkiadaki G., Decordier I., Hoek G., Merlo D.F. (2015). Outdoor air pollution exposures and micronuclei frequencies in lymphocytes from pregnant women and newborns in Crete, Greece (Rhea cohort). Environ. Res..

[128] Santovito A., Gendusa C. (2020). Micronuclei frequency in peripheral blood lymphocytes of healthy subjects living in Turin (North-Italy): Contribution of body mass index, age and sex. Ann. Hum. Biol..

[129] Rossnerova A., Spatova M., Pastorkova A., Tabashidze N., Veleminsky M., Balascak I., Solansky I., Sram R.J. (2011). Micronuclei levels in mothers and their newborns from regions with different types of air pollution. Mutat. Res. Mol. Mech. Mutagen..

[130] van Leeuwen D.M., van Herwijnen M.H.M., Pedersen M., Knudsen L.E., Kirsch-Volders M., Sram R.J., Staal Y.C.M., Bajak E., van Delft J.H.M., Kleinjans J.C.S. (2006). Genome-wide differential gene expression in children exposed to air pollution in the Czech Republic. Mutat. Res. Mol. Mech. Mutagen..

[131] Pedersen M., Wichmann J., Autrup H., Dang D.A., Decordier I., Hvidberg M., Bossi R., Jakobsen J., Loft S., Knudsen L.E. (2009). Increased micronuclei and bulky DNA adducts in cord blood after maternal exposures to traffic-related air pollution. Environ. Res..

[132] Maffei F., Hrelia P., Angelini S., Carbone F., Forti G.C., Barbieri A., Sanguinetti G., Mattioli S., Violante F.S. (2005). Effects of environmental benzene: Micronucleus frequencies and haematological values in traffic police working in an urban area. Mutat. Res. Toxicol. Environ. Mutagen..

[133] Boas D.S.V., Matsuda M., Toffoletto O., Garcia M.L.B., Saldiva P.H.N., Marquezini M.V. (2018). Workers of São Paulo city, Brazil, exposed to air pollution: Assessment of genotoxicity. Mutat. Res. Toxicol. Environ. Mutagen..

[134] Ishikawa H., Tian Y., Piao F., Duan Z., Zhang Y., Ma M., Li H., Yamamoto H., Matsumoto Y., Sakai S. (2006). Genotoxic damage in female residents exposed to environmental air pollution in Shenyang city, China. Cancer Lett..

[135] Sordo M., Maciel-Ruiz J.A., Salazar A.M., Robles-Morales R., Veloz-Martínez M.G., Pacheco-Limón J.H., Nepomuceno-Hernández A.E., Ayala-Yáñez R., Gonsebatt M.E., Ostrosky-Wegman P. (2019). Particulate matter-associated micronuclei frequencies in maternal and cord blood lymphocytes. Environ. Mol. Mutagen..

[136] Han C., Jing J.X., Sun G.X. (1997). Study of environmental pollution and damage of cytogenetic materials in urban residents. Zhonghua Liu Xing Bing Xue Za Zhi.

[137] Lemos A.T., de Lemos C.T., Coronas M.V., da Rocha J.R., Vargas V.M.F. (2020). Integrated study of genotoxicity biomarkers in schoolchildren and inhalable particles in areas under petrochemical influence. Environ. Res..

[138] Huen K., Gunn L., Duramad P., Jeng M., Scalf R., Holland N. (2006). Application of a geographic information system to explore associations between air pollution and micronucleus frequencies in African American children and adults. Environ. Mol. Mutagen..

[139] Zhao X., Niu J., Wang Y., Yan C., Wang X., Wang J. (1998). Genotoxicity and chronic health effects of automobile exhaust: A study on the traffic policemen in the city of Lanzhou. Mutat. Res. Toxicol. Environ. Mutagen..

[140] Leopardi P., Zijno A., Marcon F., Conti L., Carere A., Verdina A., Galati R., Tomei F., Baccolo T.P., Crebelli R. (2003). Analysis of micronuclei in peripheral blood lymphocytes of traffic wardens: Effects of exposure, metabolic genotypes, and inhibition of excision repair in vitro by ARA-C. Environ. Mol. Mutagen..

[141] Parry E.M., Ballantine J.A., Ellard S., Evans W.E., Jones C., Kilic N., Lewis R.I. (1997). Biomonitoring study of a group of workers potentially exposed to traffic fumes. Environ. Mol. Mutagen..

[142] Mørck T.A., Loock K.V., Poulsen M.B., Siersma V.D., Nielsen J.K.S., Hertel O., Kirsch-Volders M., Knudsen L.E. (2015). Micronucleus frequency in Danish schoolchildren and their mothers from the DEMOCOPHES population. Mutagenesis.

[143] Viegas S., Ladeira C., Costa-Veiga A., Perelman J., Gajski G. (2017). Forgotten public health impacts of cancer—An overview. Arch. Ind. Hyg. Toxicol..

[144] Schauer C., Niessner R., Pöschl U. (2004). Analysis of nitrated polycyclic aromatic hydrocarbons by liquid chromatography with fluorescence and mass spectrometry detection: Air particulate matter, soot, and reaction product studies. Anal. Bioanal. Chem..

[145] Pehnec G., Jakovljević I. (2018). Carcinogenic Potency of Airborne Polycyclic Aromatic Hydrocarbons in Relation to the Particle Fraction Size. Int. J. Environ. Res. Public Health.

[146] Birch M.E., Cary R.A. (1996). Elemental Carbon-Based Method for Monitoring Occupational Exposures to Particulate Diesel Exhaust. Aerosol Sci. Technol..

[147] Quincey P., Butterfield D., Green D., Coyle M., Cape J.N. (2009). An evaluation of measurement methods for organic, elemental and black carbon in ambient air monitoring sites. Atmos. Environ..

[148] Godec R. (2013). Temporal and Spatial Distribution of Carbon in Airborne Particulates.

[149] Conroy R.M. (2005). Stings in the Tails: Detecting and Dealing with Censored Data. Stata J. Promot. Commun. Stat. Stata.

[150] Remy A.M., Wild P. (2017). Bivariate Left-Censored Measurements in Biomonitoring: A Bayesian Model for the Determination of Biological Limit Values Based on Occupational Exposure Limits. Ann. Work Expo. Health.

