# Supplementary material for: Associating Air Pollution with Cytokinesis-Block Micronucleus Assay Parameters in Lymphocytes of the General Population in Zagreb (Croatia)
Source: Int J Mol Sci. 2022 Sep 3;23(17):10083. doi: 10.3390/ijms231710083 (PMC9455971; doi:10.3390/ijms231710083)
Supplement: Supplementary file 1 [file ijms-23-10083-s001.zip › ijms-1857871-supplementary.pdf]

Supplementary material

Associating Air Pollution with Cytokinesis-Block Micronucleus Assay Parameters in Lymphocytes of the General Population in Zagreb (Croatia)

Goran Gajski[1]*, Marko Gerić[1], Gordana Pehnec[2], Katarina Matković[1], Jasmina Rinkovec[2], Ivana Jakovljević[2], Ranka Godec[2], Silva Žužul[2], Ivan Bešlić[2], Ante Cvitković[3,4,5], Pascal Wild[6,7], Irina Guseva Canu[6], Nancy B. Hopf[6]

[1]*Mutageneisis Unit, Institute for Medical Research and Occupational Health, Zagreb, Croatia*

[2]*Environmental Hygiene Unit, Institute for Medical Research and Occupational Health, Zagreb, Croatia*

[3]*Teaching Institute of Public Health Brod-Posavina County, Slavonski Brod, Croatia*

[4]*Faculty of Dental Medicine and Health, J. J. Strossmayer University of Osijek, Osijek, Croatia*

[5]*Faculty of Medicine, J. J. Strossmayer University of Osijek, Osijek, Croatia*

[6]*Center for Primary Care and Public Health (Unisanté), University of Lausanne, Lausanne, Switzerland*

[7]*PW Statistical Consulting, Laxou, France*

***Address correspondence to:** Goran Gajski, Institute for Medical Research and Occupational Health, Mutagenesis Unit, Ksaverska cesta 2, 10000 Zagreb, Croatia; e-mail address: ggajski@imi.hr

Table S1. Model selected for the frequency of micronuclei (MNi), nucleoplasmic bridges (NPBs), nuclear buds (NBUDs) and nuclear division index (NDI) with the confounders and the first factor (PM) for 3, 7, and 30 days.

	1	3 days		7 days		30 days	
MNi		Coefficient	p-value	Coefficient	p-value	Coefficient	p-value
Year	2011	-		-		-	
	2012	-0.695	0.090	-0.696	0.093	-0.556	0.193
	2013	-0.347	0.195	-0.352	0.183	-0.502	0.030
	2014	-0.226	0.034	-0.182	0.091	-0.156	0.268
	2015	0.012	0.964	0.235	0.379	0.092	0.661
Gender	F	-		-		-	
	M	-0.168	0.067	-0.159	0.081	-0.144	0.124
Smoker	No	-		-		-	
	Yes	0.104	0.271	0.092	0.328	0.069	0.462
Temperature	<2°	-		-		-	
	2°-<10°	-0.506	0.011	-0.587	0.005	0.054	0.770
	10°-<18°	-0.138	0.463	-0.302	0.154	-0.016	0.953
	>18°	-0.069	0.761	-0.089	0.748	0.214	0.521
Factor 1 - PM		-0.579	0.082	-0.559	0.187	-0.483	0.422
NBUDs							
Year	2011	-		-		-	
	2012	-1.208	0.095	-1.213	0.094	-1.162	0.108
	2013	-0.813	0.032	-0.814	0.032	-0.284	0.252
	2014	-0.408	0.003	-0.408	0.003	-0.358	0.010
	2015	-0.371	0.274	-0.402	0.233	-0.029	0.888
Alcohol consumption	No	-		-		-	
	Yes	0.197	0.084	0.198	0.085	0.170	0.127
Factor 1 - PM		-0.185	0.539	-0.020	0.952	0.500	0.106
NPBs							
Year	2011	-		-		-	
	2012	-0.093	0.883	-0.160	0.802	-0.001	0.999
	2013	-0.894	0.147	-1.010	0.091	-0.604	0.188
	2014	-1.505	0.000	-1.380	0.000	-1.408	0.000
	2015	-0.921	0.218	-0.691	0.354	0.065	0.874
Gender	F	-		-		-	
	M	0.402	0.025	0.348	0.052	0.347	0.050
History of family cancer	No	-		-		-	

	Yes	0.498	0.014	0.519	0.005	0.373	0.042
Temperature	<2°	-		-		-	
	2°-<10°	-0.803	0.065	-0.757	0.063	0.176	0.635
	10°-<18°	0.135	0.747	-0.283	0.511	0.686	0.221
	>18°	0.347	0.485	0.113	0.833	0.923	0.163
Factor 1 - PM		-0.618	0.374	-1.244	0.117	-0.403	0.743
NDI							
	2011	-		-		-	
	2012	0.068	0.378	0.079	0.314	0.092	0.250
	2013	0.148	0.006	0.157	0.004	0.105	0.019
	2014	0.045	0.089	0.052	0.052	0.057	0.042
	2015	0.013	0.822	0.036	0.520	0.002	0.955
Factor 1 - PM		0.190	0.002	0.170	0.012	0.136	0.039

Table S2. Model selected for the frequency of micronuclei (MNi), nucleoplasmic bridges (NPBs), nuclear buds (NBUDs) and nuclear division index (NDI) with the confounders and B[a]P for 3, 7, and 30 days.

	1	3 days		7 days		30 days	
MNi		Coefficient	p-value	Coefficient	p-value	Coefficient	p-value
Year	2011	-		-		-	
	2012	-0.564	0.161	-0.565	0.160	-0.394	0.345
	2013	-0.359	0.092	-0.342	0.109	-0.417	0.059
	2014	-0.132	0.238	-0.121	0.263	-0.015	0.919
	2015	0.454	0.021	0.538	0.014	0.371	0.108
Gender	F	-		-		-	
	M	-0.169	0.062	-0.161	0.075	-0.159	0.087
Smoker	No	-		-		-	
	Yes	0.096	0.285	0.089	0.320	0.070	0.451
Temperature	<2°	-		-		-	
	2°-<10°	-0.315	0.046	-0.477	0.007	-0.045	0.804
	10°-<18°	-0.142	0.449	-0.238	0.279	-0.268	0.326
	>18°	-0.210	0.449	-0.097	0.814	-0.222	0.553
log10 - B[a]P		-0.334	0.044	-0.212	0.423	-0.470	0.046
NBUDs							
Year	2011	-		-		-	
	2012	-1.244	0.087	-1.256	0.084	-1.262	0.082
	2013	-0.308	0.228	-0.315	0.219	-0.328	0.197
	2014	-0.418	0.003	-0.421	0.003	-0.426	0.002

	2015	-0.101	0.669	-0.105	0.656	-0.126	0.579
Alcohol consumption	No	-		-		-	
	Yes	0.189	0.092	0.188	0.094	0.179	0.109
log10 - B[a]P		0.085	0.438	0.095	0.409	0.136	0.236
NPBs							
Year	2011	-		-		-	
	2012	0.117	0.847	0.148	0.811	0.093	0.882
	2013	-0.377	0.382	-0.394	0.375	-0.557	0.206
	2014	-1.291	0.000	-1.167	0.000	-1.335	0.000
	2015	0.676	0.088	0.814	0.070	0.223	0.638
Gender	F	-		-		-	
	M	0.317	0.065	0.301	0.099	0.336	0.060
History of family cancer	No	-		-		-	
	Yes	0.504	0.005	0.445	0.019	0.394	0.035
Temperature	<2°	-		-		-	
	2°-<10°	-0.455	0.162	-0.664	0.074	0.141	0.702
	10°-<18°	-0.050	0.897	-0.555	0.218	0.603	0.272
	>18°	-0.272	0.626	-0.799	0.335	0.752	0.320
log10 - B[a]P		-0.806	0.016	-1.038	0.045	-0.261	0.601
NDI							
	2011	-		-		-	
	2012	0.054	0.494	0.050	0.530	0.061	0.447
	2013	0.083	0.062	0.082	0.068	0.088	0.053
	2014	0.035	0.196	0.034	0.212	0.036	0.199
	2015	-0.051	0.293	-0.045	0.357	-0.034	0.485
log10 - B[a]P		0.057	0.007	0.055	0.016	0.050	0.036

Table S3. Method limits of detection for measured chemical species in PM_{10} samples.

Analyte	PM_{10}	$PM_{2.5}$	PM_1	OC	EC	SO_4^{2-}	NO_3^-	Cl^-	
LOD (μg/m^3)	3	2	2	0.18	0.01	0.20	0.06	0.05	
Analyte	Pb	Mn	Cd	As	Ni	Cu	Fe	Zn	B[a]P
LOD (ng/m^3)	0.05	0.05	0.001	0.004	0.18	0.15	1.6	1.1	0.035

LOD, Limit of detection
